# High genotypic diversity among methicillin-resistant *Staphylococcus pseudintermedius* isolated from canine infections in Denmark

**DOI:** 10.1186/s12917-016-0756-y

**Published:** 2016-06-29

**Authors:** Peter Damborg, Arshnee Moodley, Bent Aalbæk, Gianpiero Ventrella, Teresa Pires dos Santos, Luca Guardabassi

**Affiliations:** Department of Veterinary Disease Biology, Faculty of Health and Medical Sciences, University of Copenhagen, Stigbøjlen 4, 1870 Frederiksberg C, Denmark; Department of Veterinary Medicine, Università degli Studi di Bari, Strada P.le per Casamassima Km 3, Valenzano-Bari, 70010 Italy; Department of Biomedical Sciences, Ross University School of Veterinary Medicine, Basseterre, West Indies St Kitts and Nevis

**Keywords:** MRSP, Dog, Antibiotic resistance, Multilocus sequence typing, ST71, ST258

## Abstract

**Background:**

Methicillin-resistant *Staphylococcus pseudintermedius* (MRSP) has emerged globally in companion animals in the last decade. In Europe, the multidrug-resistant sequence type (ST)71 is widespread, but recently other clones have appeared. The objective of this study was to examine genotypic diversity and antimicrobial resistance of clinical MRSP isolates obtained from dogs, including dogs sampled on multiple occasions, in Denmark over a six-year period. For that purpose a total of 46 clinical MRSP isolates obtained from 36 dogs between 2009 and 2014 were subjected to antimicrobial susceptibility testing, multilocus-sequence typing (MLST) and SCC*mec* typing.

**Results:**

Twenty-three sequence types were identified with ST71, mostly associated with SCC*mec* II-III, as the most common occurring in 13 dogs. Among the remaining 33 isolates, 19 belonged to clonal complex (CC)258 comprising ST258-SCC*mec* IV and its single- and double-locus variants. These were susceptible to 4–7 of the 22 antibiotics tested, whereas CC71 isolates were susceptible to only 2–5 antibiotics. Clone-specific differences were especially pronounced for fluoroquinolones and aminoglycosides with most CC71 isolates being resistant and almost all CC258 isolates being susceptible. Sixteen of the 19 CC258 isolates had oxacillin MICs of 0.5 g/L, whereas MICs for CC71 isolates were consistently above 4 g/L. Four of five dogs representing multiple isolates had distinct STs on different sampling events.

**Conclusions:**

The overall genotypic diversity of MRSP is high in Denmark indicating multiple acquisitions of SCC*mec* into distinct clones, and mutational evolution, which appears to be particularly rapid for certain ancestral clones such as ST258. ST71-SCC*mec* II-III is the most common MRSP lineage and is typically multidrug-resistant. CC258-SCC*mec* IV isolates, which emerged in Denmark since 2012, display susceptibility to a wider range of antimicrobials. The isolation of distinct STs in individual dogs over time suggests repeated exposure or short-term genetic evolution of MRSP clones within patients.

## Background

*Staphylococcus pseudintermedius* is an opportunistic pathogen in dogs causing mainly integumentary infections [1]. In the last decade, methicillin-resistant *S. pseudintermedius* (MRSP) has emerged globally. MRSP is resistant to all beta-lactams, and multidrug-resistance occurs frequently, especially among multilocus sequence type (ST)71 and ST68, which predominate in dogs across Europe and North America, respectively [2]. Recently, other clones such as ST258 and closely related variants have been detected with relatively high frequency in Norway [3, 4] and the Netherlands [5], and sporadically in other EU countries [6–9]. Apart from often being resistant to fewer antimicrobial classes, these clones appear to be less virulent than ST71 as suggested by a lower tendency to produce biofilm [3] and less adherence to canine corneocytes [10]. The aim of this study was to assess the genotypic diversity and antimicrobial susceptibility of canine clinical MRSP isolates in Denmark over a 5-year period, with emphasis on temporal shifts in the general dog population and in individual dogs sampled on multiple occasions.

## Methods

A total of 46 clinical MRSP isolates obtained from integumentary infections (*n* = 37), mucosal sites (*n* = 4) or other sites (*n* = 5) were collected at the University of Copenhagen veterinary diagnostic laboratory Sund Vet Diagnostik from 36 dogs between 2009 and 2014. All isolates were verified as MRSP by *nuc* and *mecA* PCR [11, 12]. Isolates were tested for antimicrobial susceptibility using Sensititre COMPAN1F broth microdilution plates (Thermo Fisher Scientific, Hvidovre, Denmark) according to the Clinical Laboratory Standards Institute [13]. Separately for doxycycline, susceptibility was tested by disk diffusion according to the breakpoints established by Maaland et al. [14]. Isolates were also subjected to Staphylococcal Cassette Chromosome (SCC) *mec* typing [11] and multilocus sequence typing (MLST) [15]. STs were compared to the existing *S. pseudintermedius* database using the eBURST V3 software [16]. We used an arbitrary definition of clonal complexes (CCs) that included major group/subgroup founders (with more than 3 single-locus links) from the MLST database and respective single- and double locus variants (SLVs and DLVs).

## Results and discussion

A total of 23 STs were detected among the 36 dogs (Table 1), including two STs (ST258 and ST273) occurring repeatedly in individual dogs at different sampling times (Table 2). This high overall genotypic diversity is notable and resembles what was recently observed in Norway and the Netherlands [4, 5]. With regards to number of dogs infected, ST71 predominated and was detected in 13 dogs, whereas the second most common type (ST45) occurred in 3 dogs (Table 1). The frequent detection of ST71 was not surprising given its widespread appearance, and the occurrence of two closely related variants (ST270 and ST272) corroborates a recent Japanese study showing that the ST71 lineage is not as clonal as previously believed [17]. ST45 is the predominant MRSP clone in distant countries such as Australia, Thailand and Israel [18–20], but it is also relatively common in Europe. In fact, similar to our findings, it was the second most frequent MRSP clone after ST71 in both the Netherlands and in Finland [5, 21]. The eleven STs belonging to CC258 were detected in 13 dogs (Table 1). The presence of all these closely related variants of ST258 could indicate that this clone has existed and evolved for a longer time in Denmark than ST71. However, the vast majority (95 %) of ST258 isolates and variants from this study were obtained after 2012. A more plausible explanation may therefore be that the ST258 clone has a higher evolutionary rate than ST71, or that some unknown factor has favoured its recent dissemination. Interestingly, a partial and gradual replacement of CC71 with CC258 MRSP isolates was also noted in the Netherlands recently [5].

**Table 1:** Characteristics of the 46 MRSP isolates included in the study

Clonal Com-plex	MLST type	No. of isolates	No. of dogs	SCC*mec* type	No. (type) of antibiotics susceptible to^a^
CC45	ST45	3	3	NT^b^	2-3 (AMI, CHL, GEN, RIF, SXT)
	ST288^c^	1	1	V	3 (AMI, CHL, RIF)
CC68	ST271^c^	1	1	V	4 (AMI, CHL, GEN, RIF)
CC71	ST71	13	13	II-III (*n* = 11) NT (*n* = 2)	2–5 (AMI, CHL, DOX, GEN, RIF, SXT)
	ST270^c^	1	1	II–III	3 (CHL, RIF, SXT)
	ST272^d^	1	1	II–III	2 (CHL, RIF)
CC258	ST118^d^	1	1	IV	7 (AMI, CHL, ENR, GEN, MAR, RIF, SXT)
	ST258	7	2	IV	4–6 (AMI, CHL, ENR, GEN, MAR, RIF)
	ST261^c^	1	1	IV	5 (AMI, CHL, GEN, MAR, RIF)
	ST265^d^	1	1	IV	4 (CHL, ENR, MAR, RIF)
	ST267^d^	1	1	IV	6 (AMI, CHL, ENR, GEN, MAR, RIF)
	ST273^c^	2	1	IV	6 (AMI, CHL, ENR, GEN, MAR, RIF)
	ST277^c^	1	1	IV	6 (AMI, ENR, GEN, MAR, RIF)
	ST290^d^	2	2	IV, NT	6 (AMI, CHL, ENR, GEN, MAR, RIF)
	ST301^d^	1	1	IV	4 (CLI, ERY, MAR, RIF)
	ST414^d^	1	1	IV	5 (AMI, CHL, GEN, RIF, SXT)
	ST430^d^	1	1	IV	7 (AMI, CHL, ENR, GEN, MAR, RIF, SXT)
NA^e^	ST431	1	1	V	7 (AMI, CHL, CLI, DOX, ERY, GEN, RIF)
	ST268	1	1	IV	7 (AMI, CHL, ENR, GEN, MAR, RIF, SXT)
	ST269	2	2	IV	6–7 (AMI, CHL, ENR, GEN, MAR, RIF, SXT)
	ST284	1	1	V	3 (AMI, RIF, SXT)
	ST286	1	1	IV	6 (AMI, CHL, ENR, GEN, MAR, RIF)
	ST295	1	1	NT	7 (AMI, DOX, ENR, GEN, MAR, RIF, SXT)

*AMI* amikacin, *CHL* chloramphenicol, *CLI* clindamycin, *DOX* doxycycline, *ENR* enrofloxacin, *ERY* erythromycin, *GEN* gentamicin, *MAR* marbofloxacin, *RIF* rifampicin, *SXT* sulphamethoxazole and trimethoprim

^a^Out of the 22 antibiotics in the COMPAN1F broth microdilution panel. Underlined antibiotics are those for which some isolates within an ST were susceptible and others resistant

^b^NT, not typeable with the method of Kondo et al. [11]

^c^SLV of the founder of the clonal complex

^d^DLV of the founder of the clonal complex

^e^NA, not applicable as the STs are singletons

**Table 2:** MRSP clones over time in dogs that were MRSP positive on at least two sampling occasions

Dog ID	Date of isolation (month/year)	MLST type	Relatedness of clones
A	12/09	ST268	6 of 7 alleles in common (SLVs)
	08/10	ST269	
B	12/12	ST45	1 of 7 alleles in common
	01/13	ST71	
C	07/12	ST258	–
	10/12	ST258	
D	10/12	ST71	2 of 7 alleles in common
	11/12	ST295	
E	01/12	ST258	6 of 7 alleles in common (SLVs)
	03/12	ST273	
	06/12	ST273	
	07/12	ST258	
	11/12	ST258	
	12/12	ST258	
	02/13	ST258	

SCC*mec* typing confirmed the well-known association between ST71 and the SCC*mec* II-III element [2], except in two isolates where no SCC element could be identified (Table 1). SCC*mec* type IV was associated with 14 MLST types. Even though many of these types are closely related within CC258, the occurrence also in distantly related types (ST268, ST269, and ST286) suggests enhanced mobility or a low fitness cost of this element as seen in methicillin-resistant *Staphylococcus aureus* (MRSA) [22]. SCC*mec* was non-typeable in the three ST45 isolates. This may reflect presence of the pseudo-SCC*mec* element (ΨSCC*mec*_57395_) that was recently discovered in this clone by Perreten et al. [18].

Antibiotic susceptibility testing revealed the multidrug-resistant nature of MRSP with only limited susceptibility to veterinary licensed antibiotics. This was particularly pronounced for certain clones (Table 1), for example ST71 with three isolates being susceptible only to chloramphenicol and rifampicin. Isolates belonging to CC258 were generally susceptible to more antibiotic classes compared to CC71 isolates (Table 1), thus supporting the findings by Kjellman et al. [4] and Duim et al. [5]. One of the major differences was that all 15 CC71 isolates were resistant to fluoroquinolones compared to only 1 of 19 CC258 isolates (Table 1). A similar picture was observed for aminoglycosides, since 10 of 15 CC71 isolates and 2 of 19 CC258 isolates were gentamicin resistant. Interestingly, the opposite trend was evident for doxycycline, with only 8 of 15 CC71 isolates and all 19 CC258 isolates displaying resistance. Rifampicin resistance was rarely detected (1/46 isolates), thus illustrating the potential use of this drug in systemic therapy against MRSP. However, rifampicin is registered for use in humans only and should be used with caution given its potential side effects such as hepatotoxicity. Furthermore, it should not be used as monotherapy but in combination with other antibiotics, since mutations conferring resistance readily develop during rifampicin therapy [23]. A closer look at the susceptibility data showed that 16 of the 19 CC258 isolates had oxacillin MICs of 0.5 g/L, whereas MICs for CC71 isolates were consistently above 4 g/L. This could have important implications, as commercial agar plates used by some laboratories for selective isolation of MRSP were originally designed for detection of MRSA, which have a higher oxacillin breakpoint than MRSP [13] and may not allow growth of strains with low-level oxacillin resistance. In order to check this we streaked all 46 isolates on Brilliance MRSA 2 agar plates (Thermo Fisher Scientific) followed by overnight incubation at 37 °C. Only one CC258 isolate failed to grow on this medium, whereas various levels of growth were detected among remaining isolates, irrespective of their oxacillin MIC (data not shown). This indicates that at least this commercial medium has a high sensitivity for detection of MRSP, although growth may not always be as pronounced as for MRSA.

Two of the five dogs representing multiple isolates had genetically unrelated MRSP clones isolated on different sampling occasions (Dogs B and D, Table 2). Two previous longitudinal studies detected MRSP from carrier sites such as the nose and perineum for several months following MRSP infections in dogs [24, 25]. In these studies the same clone was always recovered over time in individual dogs. This difference to our study is difficult to explain, although we analysed clinical isolates and not isolates from carrier sites. Dogs B and D may have been exposed to MRSP on multiple occasions, either in their normal environment or, perhaps more likely, at the veterinary clinics they visited during infection. In Dogs A and E (Table 2), the MRSP isolates were closely related (SLVs), suggesting that these dogs might have been infected by a single clone, which later evolved within the host. In all these dogs (A, B, D, and E), infection or colonization with multiple MRSP clones at the same time is possible, since Paul et al. [26] showed high heterogeneity amongst *S. pseudintermedius* isolates originating from different body sites of the same dog. Presence of multiple clones would have gone unnoticed in this study and in many other veterinary diagnostic laboratories, which usually select only one colony representing each morphological type for susceptibility testing.

Two cases of likely dog-dog transmission were observed based on the epidemiological information we retrieved. One case concerned the two ST71 isolates with an unknown SCC*mec*. These isolates had the same susceptibility profile and originated from two unrelated dogs attending the same hospital with a 2-week interval, suggesting transmission via the hospital environment. Another case comprised two dogs living in the same family. These dogs visited a veterinary hospital two weeks apart with skin infections caused by MRSP ST71 and ST270, respectively. These STs are SLVs, and the isolates shared the same antibiotic susceptibility profile. In this case transmission between the dogs, either before or after short-term evolution of ST71, is a likely scenario, although independent acquisition of these strains cannot be ruled out.

## Conclusions

In conclusion, Danish clinical MRSP isolates are genotypically diverse indicating multiple acquisitions of SCC*mec* by distinct genetic backgrounds and rapid evolution of certain clones such as ST258. ST71-SCC*mec* II-III is the most common MRSP clone but the recent emergence of the less resistant ST258-SCC*mec* IV and related variants, signals a shift in the MRSP clonal population, which is similar to the gradual shift observed in the Netherlands recently. Further studies are needed to clarify lineage-specific virulence properties, and to assess whether the recent emergence of ST258 and related types in Europe has potential impact on veterinary care. Our results support previous evidence that MRSP can be a nosocomial pathogen [27]. Veterinary practitioners should therefore focus on hygiene measures and promote prudent antibiotic use to prevent spread of MRSP between patients.

## Abbreviations

CC, clonal complex; DLV, double-locus variant; MIC, minimum inhibitory concentration; MLST, multilocus sequence typing; MRSA, methicillin-resistant *Staphylococcus aureus*; MRSP, methicillin-resistant *Staphylococcus pseudintermedius*; SCC*mec*, Staphylococcal Cassette Chromosome *mec*; SLV, single-locus variant; ST, sequence type
